# Three‐dimensional nanoporous activated carbon electrode derived from acacia wood for high‐performance supercapacitor

**DOI:** 10.3389/fchem.2022.1024047

**Published:** 2022-10-12

**Authors:** Hamouda Adam Hamouda, Hassan Idris Abdu, Qinzheng Hu, Mohamed Aamer Abubaker, Haikuo Lei, Shuzhen Cui, Anwar I. Alduma, Hui Peng, Guofu Ma, Ziqiang Lei

**Affiliations:** ^1^ Key Laboratory of Eco-Environment-Related Polymer Materials of Ministry of Education, Key Laboratory of Polymer Materials of Gansu Province, College of Chemistry and Chemical Engineering, Northwest Normal University, Lanzhou, China; ^2^ Department of Chemistry, Faculty of Science, University of Kordofan, El Obeid, Al-Ubayyid, Sudan; ^3^ Qinba State Key Laboratory of Biological Resources and Ecological Environment, 2011 QinLing-Bashan Mountains Bioresources Comprehensive Development C. I. C., Shaanxi Province Key Laboratory of Bio-resources, College of Bioscience and Bioengineering, Shaanxi University of Technology, Hanzhong, China; ^4^ College of Life Science, Northwest Normal University, Lanzhou, China; ^5^ Department of Biology, Faculty of Education, University of Khartoum, Khartoum, Sudan

**Keywords:** supercapacitor, acacia wood, porous carbon, electrode materials, energy density

## Abstract

Herein, the novel acacia wood based hierarchical porous activated carbons (AWCs) are easily prepared, low cost and have excellent characterization, such as special biomass nanopores *via* structural stability and large specific surface areas. Activating agents such as KOH, ZnCl_2_, and H_3_PO_4_ have been used to convert acacia wood carbon into active carbons such as AWC-K, AWC-Z, and AWC-P, respectively, which are named after the activating agent. As a supercapacitor electrode, the AWC-K sample has a high yield was 69.8%, significant specific surface area of 1563.43 m^2^g^−1^ and layer thickness of 4.6 mm. Besides that, it showed specific capacitance of 224.92 F g^−1^ at 0.5 A g^−1^ in 2 M KOH as electrolyte. In addition, the AWC-K//AWC-K symmetrical supercapacitor device displays high energy density of 23.98 Wh kg^−1^ at 450 W kg^−1^ power density with excellent cycling number stability was 93.2% long lifetime of 10,000 cycles using 0.5 M Na_2_SO_4_ as electrolyte. The high electrochemistry performance mainly contributed the special biomass pores structure. Therefore, the presented approach opens new avenues in supercapacitor applications to meet energy storage.

## GRAPHICAL ABSTRACT

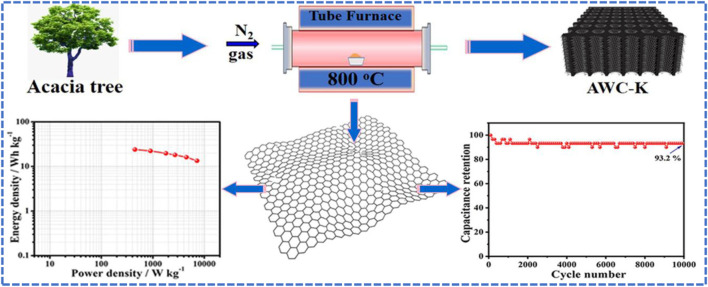

## Introduction

Due to critical environmental problems and the use of fossil fuels, one of the new natural issues confronting today’s society is the pressing need for improved energy storage systems based on green and renewable power sources (Balamurugan et al., 2019; Kalair et al., 2021). Supercapacitors (SCs) are among the most technologically advanced energy storage systems that are used in wide stationary energy storage applications like various portable and stationary electronics, hybrid electric vehicles, and used in many other advanced power devices (Chen et al., 2017; Geng et al., 2019; Leng et al., 2019; Zhang et al., 2020). SCs based on biomass-derived carbons possess the characteristics of high safety, cheap cost, and long cycling life (Peng et al., 2020; Yuan et al., 2020). Carbon-based supercapacitors cover large energy needs worldwide to enhance electrical energy storage and use on-demand (Zhang et al., 2019). The properties of the electrode materials determine how well supercapacitors perform. (Fan et al., 2015), and the types of electrodes, such as carbon materials (Yue et al., 2021), hydroxides (Ji et al., 2021; Cui et al., 2022), sulfides (Reddy et al., 2018), and transition metal oxides electrodes (Xie et al., 2019; Hamouda et al., 2022). Carbon materials, like porous carbon, carbon nanosheet, carbon fiber, carbon network, carbon nanotube and graphene are ideal electrode materials for supercapacitors due to their high surface area (Ghanem et al., 2021), premium thermal stability and high electrical conductivity (Vargheese et al., 2020). In addition, the wide distinction of biomass materials due to other diverse characteristics such as abundance, cost-effectiveness, environmental suitability, efficiency in terms of their renewability, and versatility of the selected raw materials gives a carbon-rich chemical skeleton (Wu et al., 2019; Timothy et al., 2021). Recently, many different renewable biomass carbon materials have been discovered, such as hibiscus fruits (Hamouda et al., 2021), wood-sawdust (Yang et al., 2019; Abdu et al., 2020a), fallen leaves (Li et al., 2021), baobab fruit shells (Mohammed et al., 2019), waste tea-leaves (Song et al., 2019), water bamboo (Yang et al., 2018), yeast cells (Lian et al., 2018), raw cotton (Du et al., 2018), cherry stone (Suárez and Centeno, 2020), waste paper (Durairaj et al., 2019), and human hair (Ahmed et al., 2017), have been used for energy applications. Recently, energy storage devices have been constructed using wood cells carbon materials and pseudocapacitive through chemical activation processes (Deng et al., 2016; Kostoglou et al., 2017). It should be noted that supercapacitors constructed on wood-based carbon materials have a finite specific capacitance that restricts it (Shao et al., 2020), so wood carbon chips must be activated with surface modification to obtain total capacitance (Rao et al., 2018). Many surface modification techniques used nitric acid due to the ability of the NO_3_^−^ to morphological change the surface and thus improved the electrochemical performance (Rupp et al., 2017; Zhu et al., 2017). Accordingly, after modification with nitric acid and adding an activating agent like KOH, KOH/NaOH, ZnCl_2_, FeCl_3_, KOH/FeCl_3_, and H_3_PO_4_...*etc.* Which increases the surface area and thus increases a specific capacitance for a resulting active carbon. As a result, the electrochemical performance is effective, which leads to the possibility of obtaining supercapacitors by the effect of the raise in the surface area of the material (Dong et al., 2018; Liu et al., 2018; Kim et al., 2019). *Acacia* wood sample was collected from El-Obied, Kordofan, Sudan, has low cost and no commercial value. In Sudan, acacia trees (wood) are traditionally used, where the smoke of burning wood is used to beautify women (incense), and it is also used in the manufacture of charcoal (Mohammed, 2016; MARIOD, 2019). Herein, three different kinds of activated carbon (AWCs) were synthesized from acacia wood. The AWC-K, AWC-Z, and AWC-P carbon materials were obtained from activated by KOH, ZnCl_2_, and H_3_PO_4_ agents, respectively. After the three samples were prepared and dried, the activated carbon was collected, and the yield was determined (the yield of AWC-K, AWC-Z, and AWC-P was about 69.8, 63.7, and 65.2%, respectively). The activated porous carbon of AWC-K working as an electrode in a three-electrode system with potassium hydroxide (2M) electrolyte proved good electrochemical properties as a high specific capacitance of 224.92 F g^−1^ at 0.5 A g^−1^. Besides, the AWC-K symmetric supercapacitor device, as-fabricated, displays a high energy density of 23.98 Wh kg^−1^ at a maximum power density of 450.00 W kg^−1^ and excellent cycle number stability of 93.2% retention over 10,000 cycles. Certainly, in all previous similar work, we are the first to use a high-performance supercapacitor constructed of carbon that was created *via* a chemical activation process and came from acacia wood. It has been synthesised by a chemical activation method in the presence of KOH and modified by HNO_3_.

## Experimental

### Materials

All chemicals were commercially available and used without additional purification. *Acacia* wood (collected from El-Obied, Kordofan state, Sudan), absolute ethanol (Pharmaceutical & Chemical Co., Ltd., Tianjin). Zinc chloride (ZnCl_2_, 98%) and potassium hydroxide (KOH, 99%), Industrial Corporation). Phosphoric acid (H_3_PO_4_, Sinopharm Chemical Reagent Co., Ltd., China). Nitric acid and Hydrochloric acid (HNO_3_, 99% and HCl, 99.99%, Aladdin Co., Ltd., China).

### Materials characterizations

The morphologies and structures of the AWCs materials were characterized by CuKa radiation (k = 1.5418). It was used for X-ray diffraction (XRD), which was carried out using a Rigaku D/Max-2400 diffractometer. Transmission electron microscopy (TEM, Japan), and scanning electron microscopy (FE-SEM, Germany). Raman spectra were obtained by a *Via* Raman spectrometer using an argon ion laser (Renishaw). X-ray photoelectron spectroscopy (XPS, Escalab 210 system, Germany). Atomic force microscope (AFM, NanoScope Analysis software, Digital instruments, United States). The Brunauer -Emmett-Teller (BET) surface area, before measuring nitrogen adsorption, all materials were degassed at 200°C., and the carbon samples’ pore structure was analyzed using nitrogen adsorption ASAP 2020 technology using Micromeritics’ (United States).

### Preparation of acacia wood-derived activated carbon (AWCs)

**FIGURE 1:**
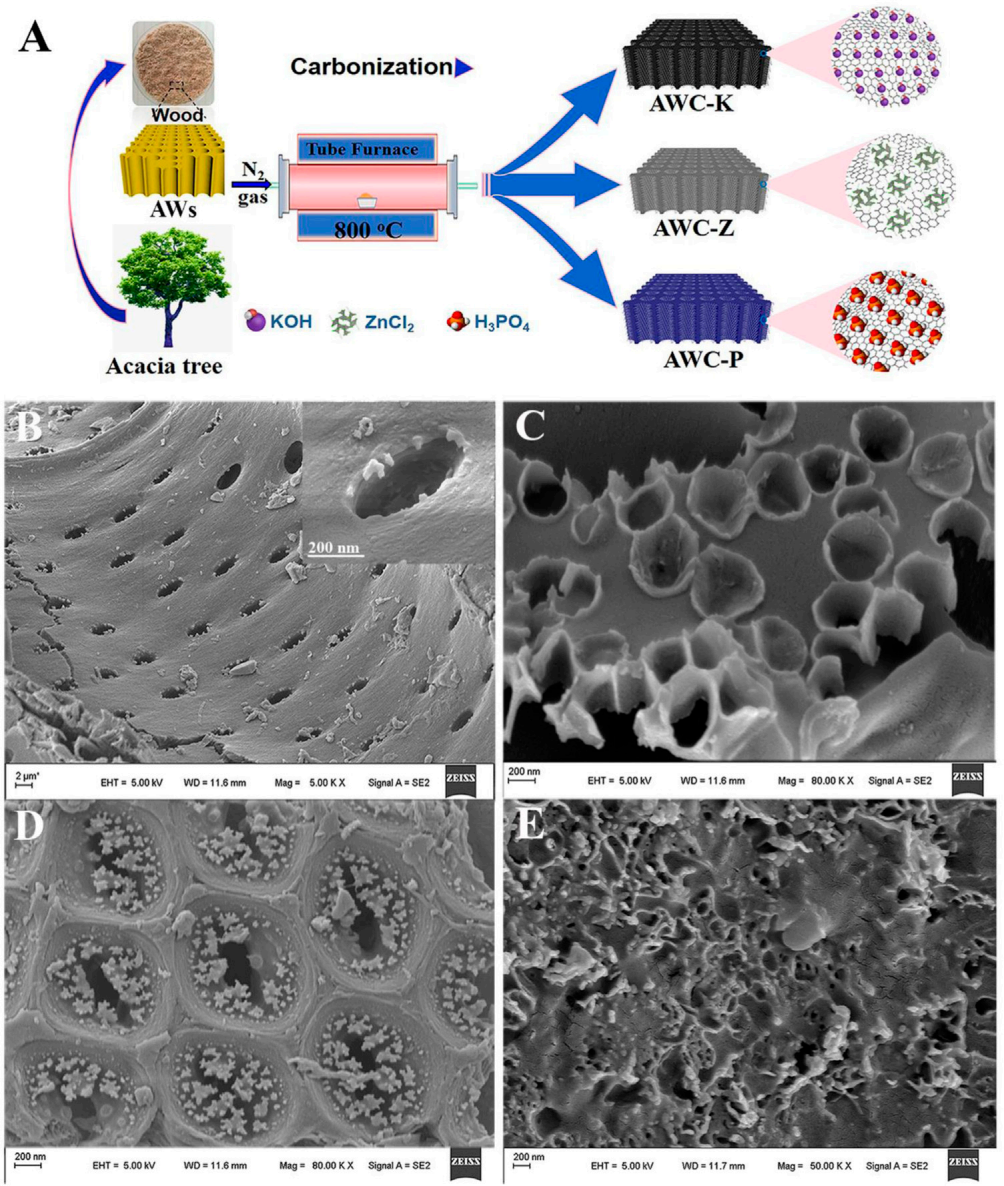
**(A)** The diagram depicting the synthesis of carbon materials from acacia wood; FE-SEM images of **(B)** AWs; **(C)** AWC-K; **(D)** AWC-Z and **(E)** AWC-P samples.

*Acacia* wood (AW) of the Sudanese was obtained from Kordofan state, Sudan. First, the (AW) was fragmented into pieces like (sawdust) and washed in deionized water, then dried at 60°C for 2 days. In a typical process, the carbon material (AWC) was obtained by pre-carbonizing the AW at 500°C for 3 h at a heating rate of 5°C min^−1^ in the N_2_ air. Then, 2.0 g of AWC was activated with 2.0 g KOH. Afterward, the above mixture was heated for 2 h in a tube furnace at 800°C with N_2_ air (called AWC-K). Subsequently, the resulting active carbon was washed with hydrochloric acid and distilled water, then dried overnight at 60°C. The AWC-Z and AWC-P samples were synthesized by the activation with ZnCl_2_ and H_3_PO_4_ agents, respectively. Using the same method as above, we prepared various carbon materials instead of using KOH as the activating agent. After that, the surfaces of the carbons were modified with an HNO_3_ solution. Figure 1A shows the formation of porous carbon materials (AWCs) from acacia wood *via* pre-carbonization and activation.

### Fabrication of the electrodes and the supercapacitor devices

A three-electrode system uses platinum (1 cm × 1 cm) as the counter electrode, saturated calomel electrode (SCE) as the reference electrode, and nickel foam as the collector. To create the working electrode, a homogenous slurry was created by combining the electrode material, the binder polyvinylidene fluoride (PVDF), and commercially available conductive carbon black in a mass ratio of 8:1:1, respectively, The slurry that was made was then put on 1.0 cm^2^ of nickel foam, dried at 60°C for 24 h, weighed, and then compressed at 15 MPa to make sheets. Each electrode’s total mass was restricted to a range of 3.0–5.0 mg. The three-electrode device was tested and validated in a 2 M Na_2_SO_4_ aqueous electrolyte. A two-electrode electrochemical cell configuration was used to realize the electrochemical performance of the supercapacitor device. Additionally, the working electrodes were made by combining the activated carbon samples, polyvinylidene fluoride (PVDF) and carbon black in a slurry with an 8:1:1 mass ratio in some drops of N-methyl pyrrolidinone solution. With the AWC-K electrode, separator, and electrolyte solution, sandwich-type cells (electrode/separator/electrode) were made.

### Electrochemical measurements

Using an electrochemical workstation (CHI 660D) for galvanostatic charge-discharge (GCD), cyclic voltammetry (CV), and electrochemical impedance spectroscopy (EIS) tests, the electrochemical performance of all electrode materials (AWCs) was evaluated. And the cycling testing equipment was used to perform the cycle-life stability test (CT 2001A). CV and GCD measures were carried out at different scan rates and current densities in a sufficient potential window. The GCD curves determined the particular capacitance of each as-prepared working electrode using Eq. 1 below.

$$C_{m}=I\;\Delta t\;/\;\left(m\;\Delta V\right)$$ (1)

Where, C_m_ (F g^−1^) ≡ specific capacitances, I (A) ≡ discharge current, Δt (s) ≡ time of discharge, m (g) ≡ weight of active material, and ΔV (V) ≡ potential window.

The electrochemical performance of the AWC-K//AWC-K device*,* and the specific capacitance of a fabricated supercapacitor device was determined using Eq. 1. Using the following Eqs 2,3, we were able to get the energy density (Wh kg^−1^) and power density (W kg^−1^) of the two-electrode cell.

$$E=C_{m}\times \Delta V^{2}/7.2$$ (2)

P=3600 E /Δ (3)

## Results and discussion

### The morphology and texture characterization

FE-SEM images were acquired to verify the developments and effects of carbon materials. Figure 1B shows SEM images of the (AWs), which have micropores that are uniformly and symmetrically distributed and show a rough surface. This indicates that the carbon skeleton is relatively stable, and can be used as a natural model in energy applications. Figure 1C shows the product regulated by AWCs and KOH model, such as AWC-K, exhibits a die-like shape (AWs), and displays a clear porous structure. Due to their large surface areas, hierarchical pore structures, or superior conductivity to speed up the transfer of electrolyte ions, we note that the hierarchical carbon materials of AWC-K porous carbons (PCs) are frequently used as a supercapacitor electrode. In order to optimize their electrical performance, *p*Cs feature porous structures, such as microporous or mesoporous structures based on how the pore structures of the electrode materials affect the charge storage mechanism of EDLCs (Xing et al., 2018). The mesoporous/microporous material increases the specific surface area and offers plenty of electrolyte ion adsorption sites. Additionally, mesopores have shorter diffusion paths, enhancing the electrodes’ electrical efficacy (Song et al., 2018). As a result, we conclude that AWC-K features porous carbon electrodes that are advantageous for electrochemical capacitor charge and storage.

Figure 1D shows the mesopores and macropores are randomly distributed on the surface of the carbon structure and macro internal cracks due to adding activation reagent (ZnCl_2_). Figure 1E displays the carbon nanostructures and nanospheres, which are irregularly distributed on the carbon structure caused by the effect of (H_2_PO_3_) on the model AWCs to give AWC-P.

**FIGURE 2:**
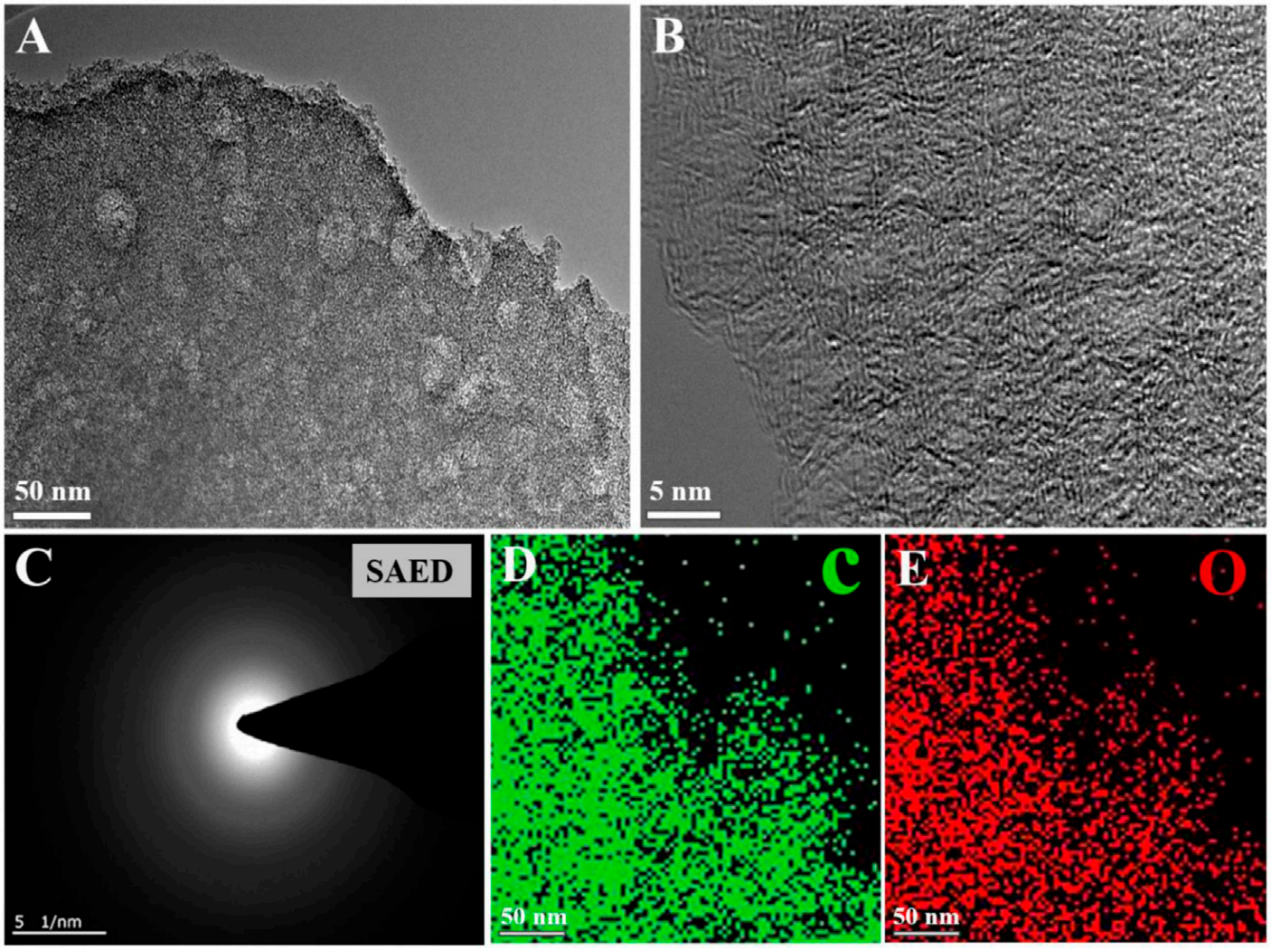
**(A)** TEM image of AWC-K; **(B)** HR-TEM image of AWC-K; **(C)** SAED pattern of AWC-K; **(D–E)** the images of the different AWC-K elements that are distributed.

FE-TEM was also introduced to study the sample’s porous texture. The TEM image of AWC-K suggests a thin smooth surface with small spots and wrinkles in the center and pleated silk-like edges (Figure 2A). As shown in Figure 2B, a magnified image of the carbon nanopores, which shows a very rough surface to form an interconnected structure of good character in energy storage applications. In addition, the AWC-K material provided the SAED pattern (Figure 2C), demonstrating the characteristic carbon nature of the sample. The 2 C and O atoms were evenly distributed throughout AWC-K (Figure 2(D-E)), according to the results of the TEM element mapping investigation.

**FIGURE 3:**
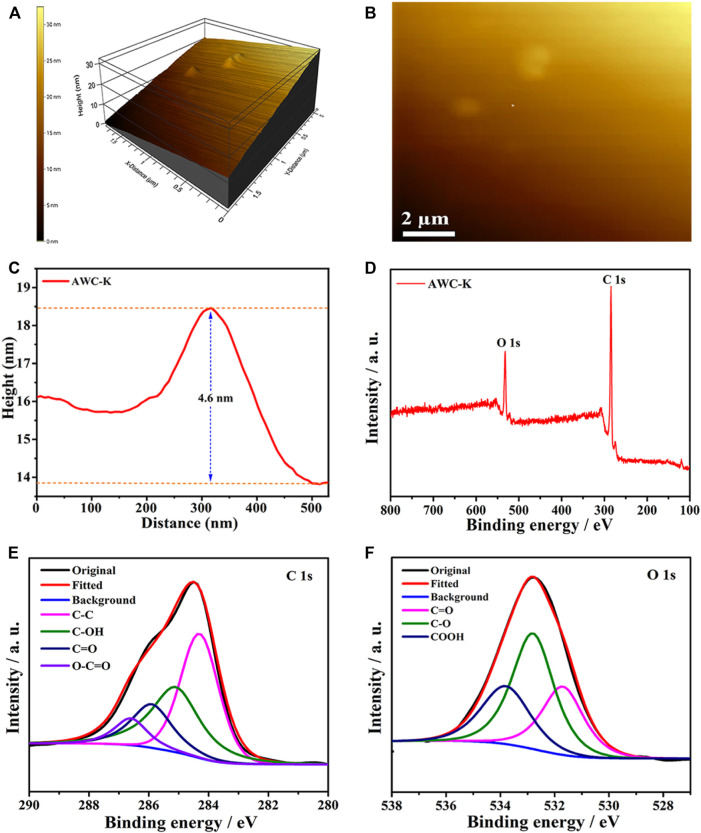
**(A,B)** AFM images of AWC-K; and **(C)** The thickness curve of AWC-K; **(D)** XPS survey spectrum, HR- XPS spectra of C 1s **(E)** and O 1s **(F)** of AWC-K.

Figure 3(A-B) shows the atomic force microscopy (AFM) amplitude three-dimension and two-dimension images of the AWC-K surface, respectively. The measurements resulting from different areas of a carbon surface confirm that the thickness of the AWC-K is about 4.6 nm (Figure 3C). AFM revealed smooth spherical surface morphologies as shown in Figure 3A. Moreover, the AWC-K sample showed slight variation in its size distribution. The different ways carbon atoms get attached to each other can explain why AWC-K has a broader range of sizes.

The structure of AWCK was further confirmed using X-ray photoelectron spectroscopy (XPS) analysis; the survey spectrum (Figure 3D) displays two peaks at a binding energy of 285 and 532 eV, properties that apply to C1s and O1s orbits, respectively, this suggests the presence of C and O elements in the AWC-K sample. Besides, there are no impurities in the range of 200–800V, which indicates the high purity of AWC-K. The proportion of oxygen and carbon elements in AWC-K was determined from the XPS survey spectrum to be about 84.72% and 15.28%, respectively. Mainly, four carbon species compositions were detected in the spectrum of C1s, such as sp^3^ graphite carbon (284.4 eV), Csp^2^-C-OH (285.2 eV), Csp^3^-O (285.96 eV), and C = O (286.95 eV) (Figure 3E), which detection the carbon atoms activated by KOH. There are three main curves featured in the O1s level of AWC-K (Figure 3F). Typically attributed to C = O in carboxylic acid/ketone (531.7 eV), O-C in epoxy/phenol/ether, lactone (532.8 eV), C = O for carboxylic acid (533.8 eV) (Peng et al., 2019b). Practical charge storage surface areas are obtained by wettability with the aqueous electrolyte, which depends on the C-O and C = O types.

**FIGURE 4:**
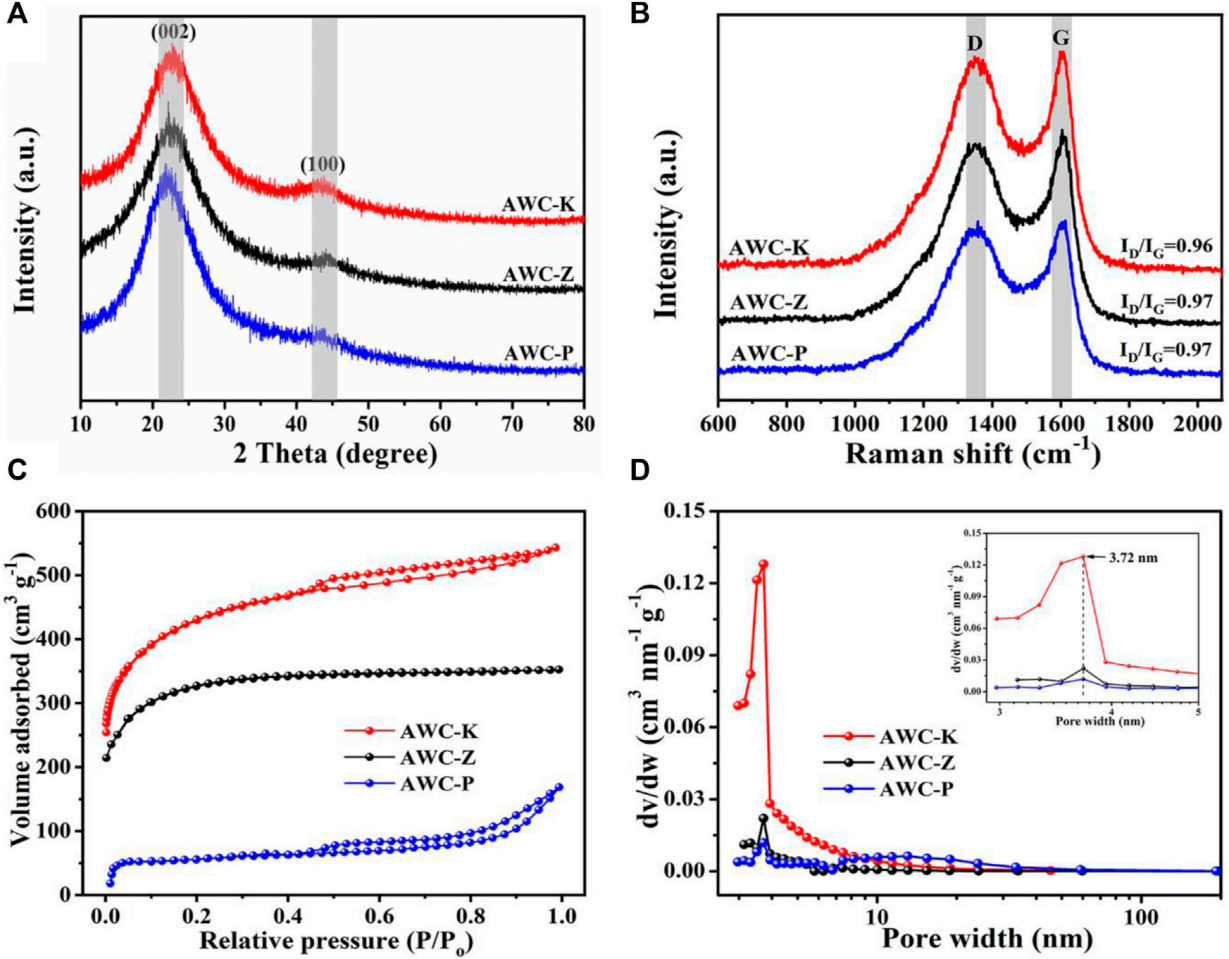
**(A)** XRD patterns of the AWC-K, AWC-Z, AWC-P; **(B)** Raman spectrum of the AWC-K, AWC-Z, AWC-P; **(C)** Nitrogen sorption isotherms and **(D)** pore size distribution curves of the samples.

The crystal structure was further studied and investigated after activation processes prepared carbon samples (AWCs) using the XRD patterns shown in Figure 4A. The carbon samples obtained from different activations agents show similar XRD patterns with two broad peaks at about 43 and 23 corresponding to the (100) and (002) lattice planes. This suggests that randomly forming carbon layers are what give amorphous activated carbon its turbulent structure with nanopores that serve to increase the specific surface area (Yin et al., 2020). This analysis suggests that the activation process with the activation agents as KOH, ZnCl_2_, and H_3_PO_4_ confirms that the AWCs have been converted into graphitic carbon and amorphous carbon with good electrochemical properties.

Raman spectroscopy is one of the basic analytical processes for identifying graphitic carbon structure and proving activation in carbon-based materials. The peaks in the Raman spectra of the activated carbons were identified as being around 1600 and 1350 cm^−1^ for the G-band and D-band carbon structure, respectively (Figure 4B). The I_D_/I_G_ ratio of AWCs is smaller than 1, which suggests that the stability of AWCs is long-range with the structure ordered. The G and D bands suggest that the as-prepared samples have an amorphous quality, which conforms to the XRD data.

To confirm the 3D structure of the biomass-derived active carbons (AWCs), the N_2_ adsorption/sorption isotherm was performed to consider the optimization and activation of the carbon structures by H_3_PO_4_, ZnCl_2_, and KOH at constant temperatures of 800°C are shown in Figure 4C. Isothermal type I, typical of microporous materials, is present in all varieties of porous carbon. The porous character and narrow pore size distribution are seen in the apparent rise in the adsorbed volume corresponding to low relative pressure (P/P0) (Abdu et al., 2020b). It investigated how activating agents affected the porosity of carbon samples. The specific surface area of AWC-K, AWC-Z, and AWC-P was 1563.43, 1210, and 205.36 m^2^ g^−1^, respectively. Therefore, the highest porosity development was observed for the sample activated by KOH. The porous carbon’s pore size distribution was derived from acacia wood was estimated using a BJH model is shown in Figure 4D. Revealed that all prepared samples (AWC-K, AWC-Z, and AWC-P) had a hierarchically developed pore structure consisting of micropores and mesoporous. It can be concluded that the activated carbon has a low resistance due to the porosity, this makes it possible for ions to diffuse quickly and thus improves the electrochemical behavior. The most popular method for determining the porosity of materials having mesopores (pore widths between 2–50 nm) is gas physisorption. Using the adsorption data and an appropriate mathematical model, the pore size distribution, pore volume, and pore area may be determined. Although this method can be used to evaluate samples with pores up to 300 nm in size, it is most effective for pores between 2–100 nm when Types II or IV isotherms are obtained. Measuring mesopores is based on the same basic ideas and steps as BET surface area analysis with the static volume method (Chen et al., 2021). Measuring mesopores is based on the same basic ideas and steps as BET surface area analysis with the static volume method. Pore size can be calculated using the pore volume as follows: A = 2 V/r, where A is the surface area, V is the pore volume, and r is the pore radius. The structural parameters of the AWC-derived carbon materials are illustrated in Table 1

**TABLE 1:** Yield, Textural parameters, and raman tests of samples.

Sample	Yield (wt%)	S_BET_ ^a^ (m^2^ g^−1^)	S_mic_ ^b^ (m^2^ g^−1^)	D^c^ (nm)	V_total_ ^d^ (cm^3^ g^−1^)	I_D_/I_G_ ^e^
AWC-K	69.8	1563.43	163.68	3.72	0.832	0.96
AWC-Z	63.7	1210.00	021.15	3.74	0.546	0.97
AWC-P	65.2	0205.36	045.18	3.71	0.261	0.97

a Specific surface area was determined using the BET, method.

b Micropore surface area from t-plot method.

c Average pore diameter.

d Total pore volume at P/P_0_ = 0.99.

e The intensity ratio of the D band to the G band.

### The electrochemical performance of the three-electrode

**FIGURE 5:**
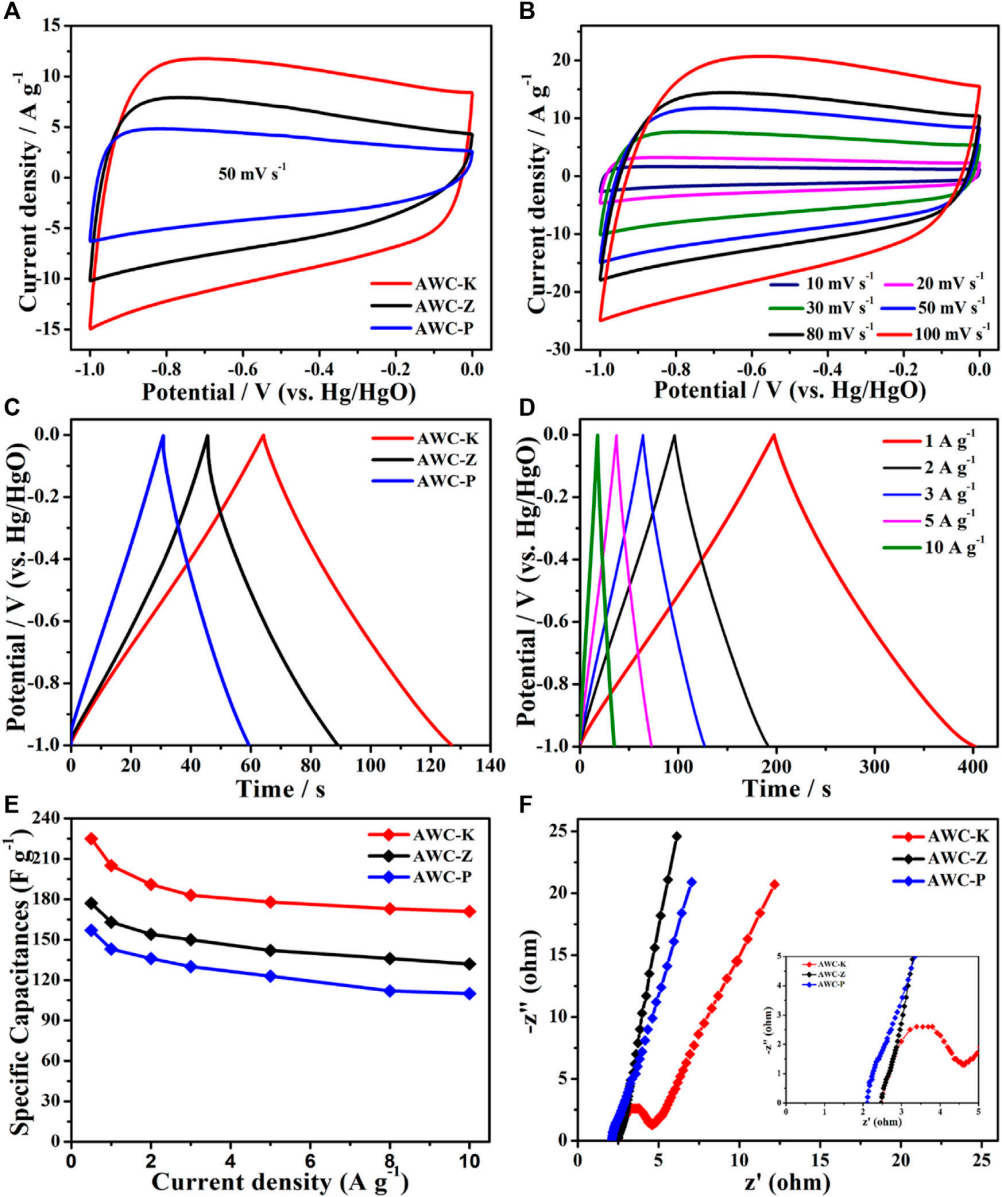
**(A)** CV curves of AWC-K, AWC-Z and AWC-P electrode at 50 mV s^−1^; **(B)** CV curves of AWC-K electrode; **(C)** GCD curves of AWC-K, AWC-Z and AWC-P at 3 A g^−1^; **(D)** GCD curves of AWC-K at various current densities; **(E)** Specific capacities of the AWC-K, AWC-Z and AWC-P electrode at different current densities; **(F)** The Nyquist plots of the AWC-K, AWC-Z and AWC-P electrode.

The electrochemical behavior of AWCs was studied by a three-electrode cell using a 2 M KOH electrolyte. The cyclic voltammetry (CV) curves of AWC-K, AWC-Z, and AWC-P electrodes are shown quasi-rectangular shapes (Figure 5A), indicating that these electrodes have perfect capacitive behavior and fast electrochemical response (Nirmaladevi et al., 2021). The CV curve of the AWC-P electrode has a triangular orientation at a high potential, due to the small pore size and slow electrolyte diffusion, which leads to the effect of electrode ions moving and thus low electrochemical properties (Sun et al., 2018; Peng et al., 2019a). From the CV curve, it is noted that the area of the AWC-K electrode is more significant than that of another electrode that scan at the same rate (50 mV s^−1^). This suggests that it has a more significant specific capacitance than AWC-Z and AWC-P electrodes. Figure 5B shows the CV curve of AWC-K with different scan rates, the curve is slightly deformed as the area increases. Still, it maintains a consistent rectangular shape when the scanning rate is increased, proving the AWC-K electrode has a high ability to transfer ions and electrons rapidly through the electrolyte solution. The galvanostatic charge/discharge (GCD) test is crucial to proving the AWC-K electrode’s performance in capacitance. The GCD curves of AWCs electrodes at 3 A g^−1^ are shown in Figure 5C. The symmetrical triangle-like shapes indicate that the storage process is predominantly dominant for AWC-K, AWC-Z, and AWC-P electrodes. The GCD curves of AWC-K show the typical symmetric triangle shape with different current densities (Figure 5D), which suggests that the AWC-K electrode has remarkable electrochemical reversibility. Therefore, Eq. 1 shows how the GCD curves were used to figure out the specific capacitances of the electrodes (Lei et al., 2020). Figure 5E shows the specific capacitance for the electrode materials and their corresponding various current densities. It should be noted that at the same current density of 0.5 A g^−1^, AWC-K has a specific capacitance of 224.92 F g^−1^, which is higher than that of AWC-Z at 177 F g^−1^ and AWC-P at 157.1 F g^−1^ Figure 5F displays the Nyquist plots of AWC-K, AWC-Z and AWC-P electrodes. One can see that the Nyquist plots of AWCK were given a smaller semicircle in a high-frequency zone and a more minor real axis (Z′) intercept for AWC-K. The nanopores AWC-K material, which makes it easier for the electrolyte to move around and diffuse, is responsible for the electrochemical performance, which leads to high rate capacitance.

### Electrochemical performance of the two-electrode

**FIGURE 6:**
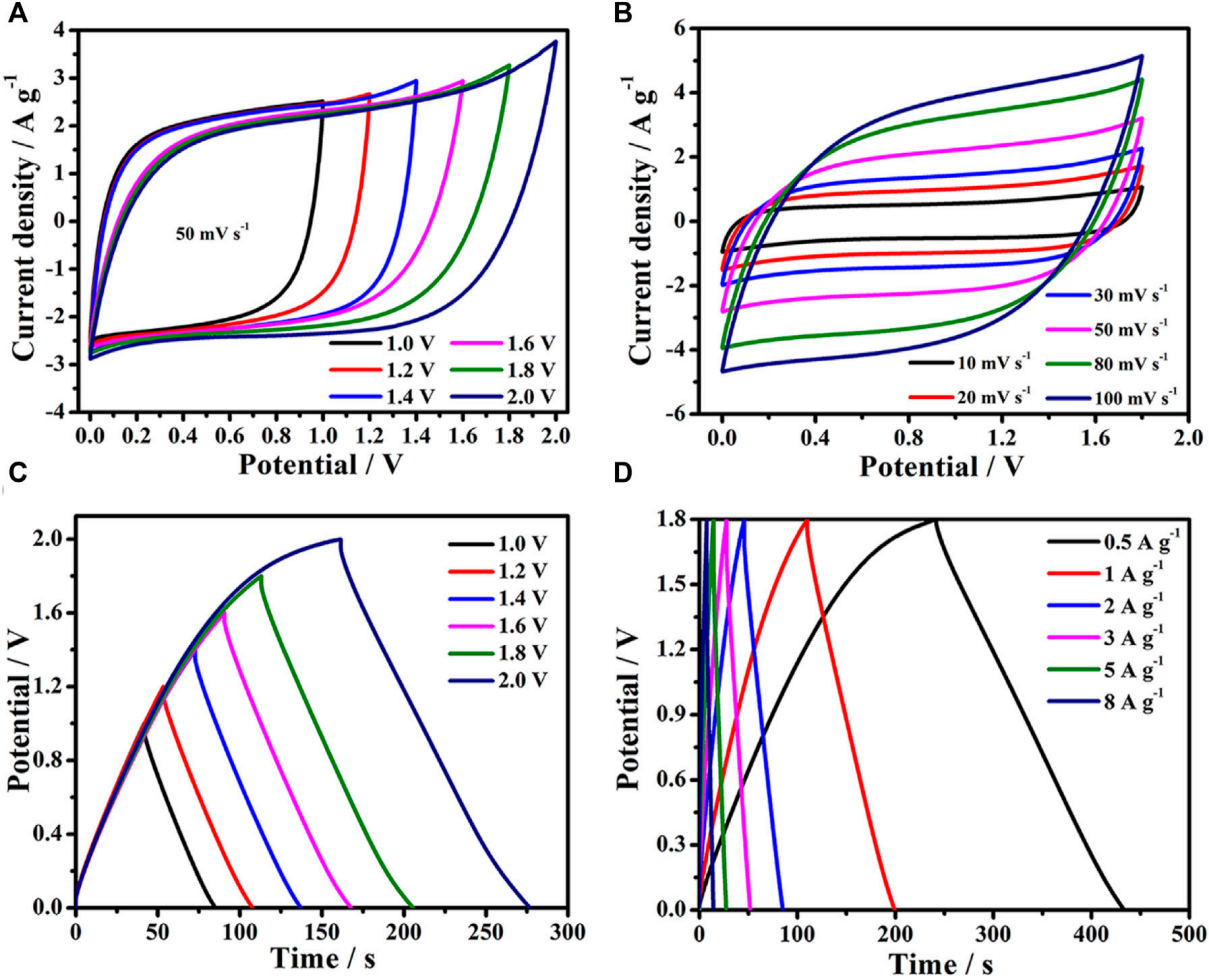
**(A)** CV curves of the AWC-K//AWC-K device at 50 mV s^−1^ with various voltage; **(B)** CV curves of the two-electrode cell at different scan rates; **(C)** GCD curves of the two-electrode cell at different current densities; **(D)** The GCD curves of the two-electrode cell at 1 A g^−1^.

To confirm the practicality of the AWC-K electrode, the electrochemical performance of AWC-K//AWC-K was tested with complement voltage operated in an aqueous KOH electrolyte was investigated in a cell device. The electrochemical performance of AWC-K electrodes is used in a supercapacitor device with a symmetrical design and a broad operating voltage. Therefore, the CV tests were performed at a constant scan rate of 50 mV s^−1^ in various voltage windows of 1.0, 1.2, 1.4, 1.6, 1.8, and 2.0 V, to verify the operating voltage range of the AWC-K//AWC-K device (Figure 6A). The symmetric supercapacitor device’s CV curves were given an asymmetric quasi-rectangular shape, from a minimum scanning rate of 10 mV s^−1^ to a maximum scanning rate of 100 mV s^−1^ at 1.8 V, as shown in Figure 6B. The GCD curves of the AWC-K//AWC-K device at different voltage ranges suggest that there is perfect electrochemical reversibility. Therefore, the operating voltage is measured in the range (1.0–2.0 V) with 1 A g^−1^. The best voltage to calculate the GCD test for supercapacitors is determined (Figure 6C). The typical GCD curves of the AWC-K//AWC-K device with various current densities (0.5–5 Ag^-1^) at 1.8 V (Figure 6D). It is noted that all the GCD curves are the charge and discharge curves almost symmetric and like an isosceles triangle. These findings suggest that a symmetric cell has very good electrochemical reversibility and possesses ideal capacitive behaviors.

**FIGURE 7:**
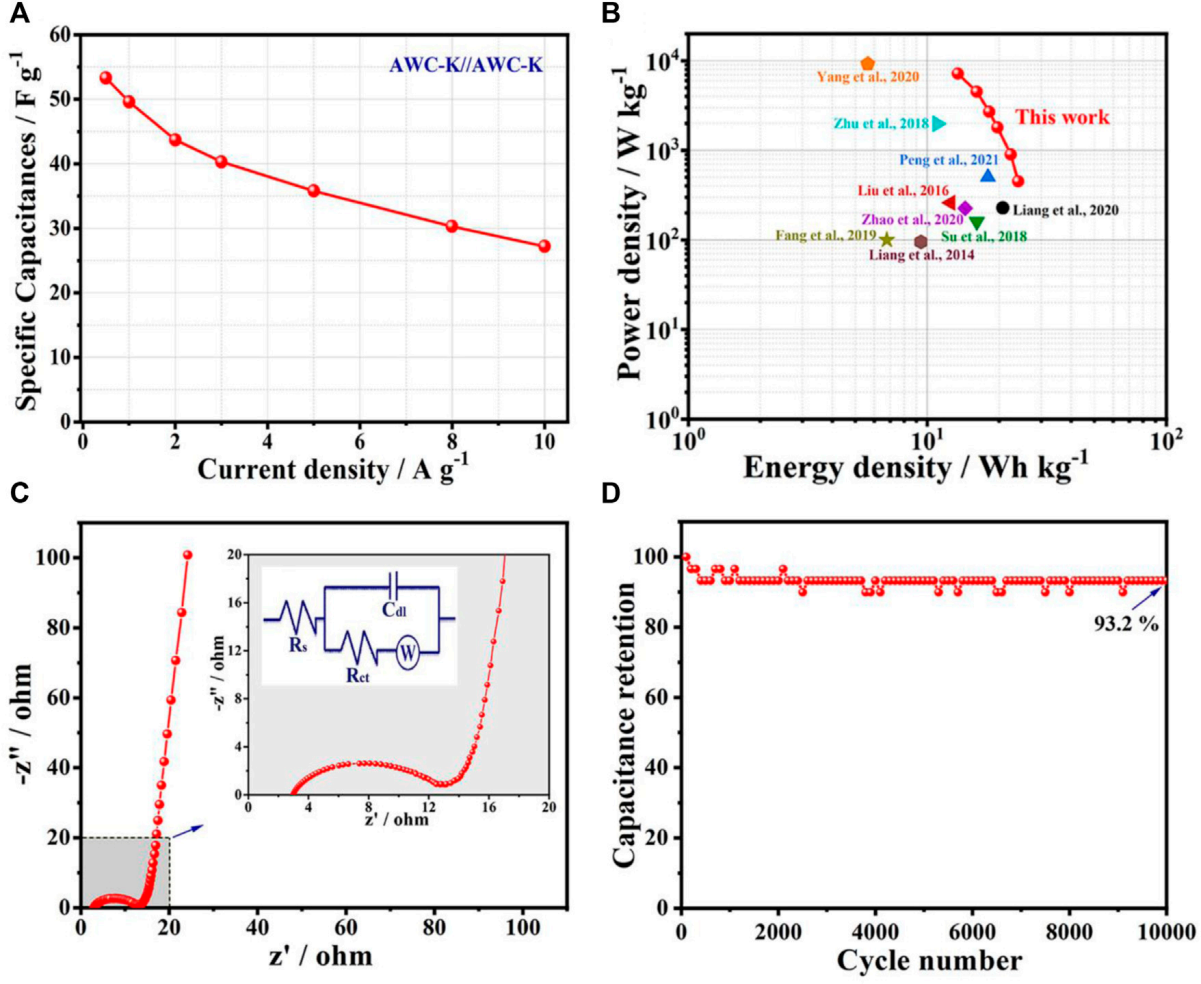
**(A)** Specific capacities of the AWC-K//AWC-K device at various current densities; **(B)** Ragone plots of the AWC-K//AWC-K device and; **(C)** Nyquist plots of the AWC-K//AWC-K device (the inset of the equivalent circuit of EIS); **(D)** The cycling stability of the symmetric cell at 3 A g^−1^ in 0.5 M Na_2_SO_4_ electrolyte.

When various current densities are applied to the specific capacitances of the SSC device, it is clear that the specific capacitances proclivity drops as the current density rises. The specific capacities at 0.5, 1, 2, 3, 5, 8, and 10 A g^−1^ were observed to be 53.3, 49.6, 43.7, 40.3, 35.8, 29.8, and 27.2 F g^−1^, respectively. As the charge and discharge curves are the same shape over time as the current density goes up, the discharge curves are almost the same shape as their parallel counterparts (Figure 7A). The specific capacitance for symmetric supercapacitor is used to determine the energy and power densities in the Ragone plot of AWC-K//AWC-K, revealed that a high energy density of 23.98 Wh kg^−1^ at the 450 W kg^−1^, and 13.41 Wh kg^−1^ with 7200 W kg^−1^ power density (Figure 7B). The energy density of SSC in this work (AWC-K//AWC-K) was calculated to be higher than that of other similar supercapacitors, such as LMCN-2//LMCN-2 (20.7 Wh kg^−1^) (Liang et al., 2020), N-HCNs-800//N-HCNs-800 (17.92 Wh kg^−1^) (Peng et al., 2021), SAC-4//SAC-4 (16.1 Wh kg^−1^) (Su et al., 2018), HPC-700//HPC-700 (14.40 Wh kg^−1^) (Zhao et al., 2020), LSC800//LSC800 (12.5 Wh kg^−1^) (Liu et al., 2016), AC1//AC1 (11.00 Wh kg^−1^) (Zhu et al., 2018), HLPC//HLPC (9.4 Wh kg^−1^) (Liang et al., 2014), HPCR-800//HPCR-800 (6.77 Wh kg^−1^) (Fang et al., 2019), and CS-H3//CS-H3 (5.63 Wh kg^−1^) (Yang et al., 2020), see (Table 2).

**TABLE 2:** Comparison of symmetric cells’ electrochemical performance in the references.

Symmetric device	Cycling stability	Energy density (wh kg^−1^)	Power density (W kg-1)	References
AWC-K	93.2% after 8000 cycles	23.98	450	[This work]
LMCN-2	90.7% after 10,000 cycles	20.70	228	Liang et al. (2020)
N-HCNs-800	90.0% after 20,000 cycles	17.92	500	Peng et al. (2021)
SAC-4	81.3% after 10,000 cycles	16.10	160	Su et al. (2018)
HPC-700	93.0% after 15,000 cycles	14.40	225	Zhao et al. (2020)
LSC800	95.4% after 10,000 cycles	12.50	260	Liu et al. (2016)
AC1	92.0% after 10,000 cycles	11.00	1980	Zhu et al. (2018)
HLPC	98.0% after 1000 cycles	9.40	95	Liang et al. (2014)
HPCR-800	81.0% after 10,000 cycles	6.77	100	Fang et al. (2019)
CS-H3	99.8% after 10,000 cycles	5.63	9213	Yang et al. (2020)

The electrochemical properties of the SSC device were discovered using electrochemical impedance spectroscopy (EIS) (Rehman et al., 2022). Figure 7C shows that the charge transfer resistance (Rct = 12.98), which was used to make the electron double-layer capacitor (C_dl_), is typified by a modest resistance (Rs = 2.95), semicircle line in the high-frequency zone. Additionally, the approximately straight line in the low-frequency area shows the Warburg resistance (W). Due to fast ion diffusion and transport lowers impedance and the interaction between the electrode and the electrolyte (Cui et al., 2021).

The cycling stability of the AWC-K//AWC-K SSC device is 93.2% with the capacitance retention after 10,000 cycles at 5 A g^−1^ (Figure 7D), showing excellent long-term stability of this activated carbon electrode. Furthermore, an essential characteristic of high-performance supercapacitors is cyclic stability.

## Conclusions

In brief, novel acacia wood hierarchical porous activated carbons (AWCs) were prepared from acacia wood using KOH, ZnCl_2_, and H_3_PO_4_ as chemical activation agents during carbonization processes. Due to its large specific surface area, the AWC-K used as the supercapacitor electrode provides a significant specific capacitance. Besides, the AWC-K electrode-based symmetric supercapacitor device exhibits a high energy density of 23.98 Wh kg^−1^ at 450 W kg^−1^ and superior cycle stability of 93.2% capacitance retention rate. Therefore, acacia wood’s utilization in supercapacitors applications can meet energy storage needs. These results suggest that AWC-K as an electrode possesses good carbon-based properties, which are expected to be useful in electrochemical energy storage system applications.

## Data Availability

The original contributions presented in the study are included in the article/supplementary material, further inquiries can be directed to the corresponding author.
